# Accurate prediction of nucleic acid binding proteins using protein language model

**DOI:** 10.1093/bioadv/vbaf008

**Published:** 2025-01-20

**Authors:** Siwen Wu, Jinbo Xu, Jun-tao Guo

**Affiliations:** Department of Bioinformatics and Genomics, University of North Carolina at Charlotte, Charlotte, NC 28223, United States; Toyota Technological Institute at Chicago, Chicago, IL 60637, United States; Department of Bioinformatics and Genomics, University of North Carolina at Charlotte, Charlotte, NC 28223, United States

## Abstract

**Motivation:**

Nucleic acid binding proteins (NABPs) play critical roles in various and essential biological processes. Many machine learning-based methods have been developed to predict different types of NABPs. However, most of these studies have limited applications in predicting the types of NABPs for any given protein with unknown functions, due to several factors such as dataset construction, prediction scope and features used for training and testing. In addition, single-stranded DNA binding proteins (DBP) (SSBs) have not been extensively investigated for identifying novel SSBs from proteins with unknown functions.

**Results:**

To improve prediction accuracy of different types of NABPs for any given protein, we developed hierarchical and multi-class models with machine learning-based methods and a feature extracted from protein language model ESM2. Our results show that by combining the feature from ESM2 and machine learning methods, we can achieve high prediction accuracy up to 95% for each stage in the hierarchical approach, and 85% for overall prediction accuracy from the multi-class approach. More importantly, besides the much improved prediction of other types of NABPs, the models can be used to accurately predict single-stranded DBPs, which is underexplored.

**Availability and implementation:**

The datasets and code can be found at https://figshare.com/projects/Prediction_of_nucleic_acid_binding_proteins_using_protein_language_model/211555.

## 1 Introduction

Nucleic acid binding proteins (NABPs), including DNA binding proteins (DBPs) and RNA binding proteins (RBPs), play crucial roles in many biological processes, such as DNA replication and repair, transcriptional regulation, alternative splicing and translation (Luscombe *et al.* 2000, Gerstberger *et al.* 2014, Hudson and Ortlund 2014). There are two types of DBPs, single-stranded DNA (ssDNA) binding proteins (SSBs) and double-stranded DNA (dsDNA) binding proteins (DSBs). SSBs are mainly involved in DNA recombination, replication and repair, and serve as key players in the maintenance of genomic stability, while DSBs participate in transcriptional regulation, DNA cleavage and chromosome packaging (Corona and Guo 2016, Lin *et al.* 2021, Guo and Malik 2022). Computational prediction of NABPs has been considered an efficient alternative to the expensive and time-consuming experimental methods for functional annotation of the vast number of uncharacterized proteins in the protein database.

**Figure 1.**
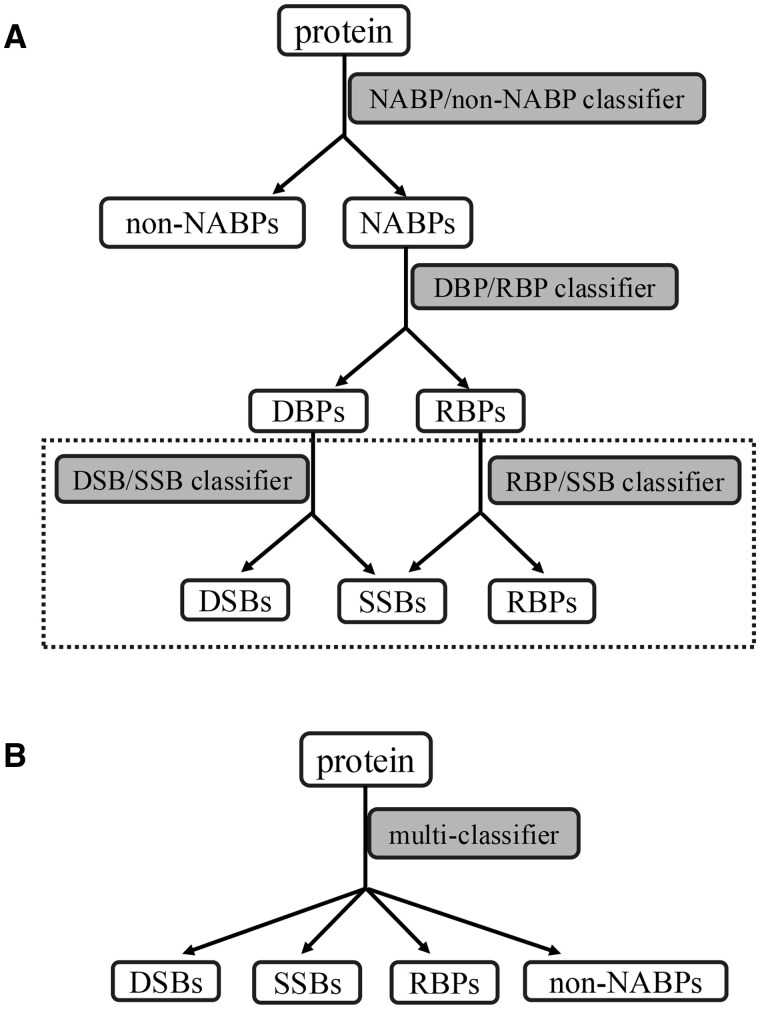
Flowchart of the hierarchical (A) and multi-class (B) approaches. The classifiers in the dashed box are described in a recent study (Wu *et al.* 2024).

Many DBP and RBP predictors have been developed so far with classical machine learning based models such as Support Vector Machine (SVM) and Random Forest (RF), and more advanced deep learning approaches including Convolutional Neural Network (CNN) using sequence-based features to train and test the models (Kumar *et al.* 2007, Lou *et al.* 2014, Xu *et al.* 2014, Motion *et al.* 2015, Chowdhury *et al.* 2017, Qu *et al.* 2017, Zhang and Liu 2017, Rahman *et al.* 2018, Zheng *et al.* 2018, Adilina *et al.* 2019, Ali *et al.* 2019, Du *et al.* 2019, Hu *et al.* 2019, Mishra *et al.* 2019, Wang *et al.* 2020, Zhang *et al.* 2021, Pradhan *et al.* 2023a,b). Most of these predictors only target one type of NABPs, either DBPs or RBPs, which limits their application in predicting the types of NABPs for proteins without known functions. Recently we developed a hierarchical approach and a multi-class approach for prediction of NABP types for any given protein using a combination of deep learning methods and a sequence-based feature, position specific scoring matrix (PSSM) (Wu and Guo 2024). While our DSB/RBP predictor outperforms published models and demonstrates a balanced prediction between the positive and negative datasets, the overall prediction accuracy is modest at 72% for any given protein. Moreover, for the first time, we explicitly included annotated SSBs as part of the DBPs dataset in that study and investigated the prediction accuracy of SSBs and their effect on the overall prediction accuracy. We found that the accuracy for SSBs is only about 40% with over half of them predicted as RBPs (Wu and Guo 2024). In the past several years, machine learning models have been developed for classification between SSBs and DSBs (Wang *et al.* 2017, Tan *et al.* 2019, Ali *et al.* 2020, Sharma *et al.* 2021, Manavi *et al.* 2023). Based on the dataset developed by Wang *et al.* (2017), the prediction accuracy for SSB/DSB classification ranges from 73% to about 83%, with Manavi *et al.* reported a higher accuracy using a CNN method and evolution-based features (Manavi *et al.* 2023). However, these SSB/DSB classification models assume each input protein is a known DBP. Therefore, the usefulness of these methods is limited as we demonstrated previously that the major issue is the wrong prediction of SSBs as RBPs. To address this issue and to improve the prediction accuracy of SSBs, we recently developed an improved SSB/DSB classifier as well as a novel SSB/RBP classifier (dashed box in Fig. 1A) with a pretrained protein language model ESM2 and we demonstrated high prediction accuracy for both SSB/DSB and SSB/RBP classifiers (Wu *et al.* 2024).

In this work, we aim to develop models for improving prediction accuracy of different types of NABPs, including SSBs, for any given protein by taking advantage of the datasets we generated in our previous study and the powerful protein language model ESM2 (Lin *et al.* 2023). The major differences between this work and our previous studies lie in two aspects. Unlike our previous study that evaluated the prediction accuracy of SSBs as part of the DBP dataset since the small SSB dataset is not suitable for deep learning approaches, here we explicitly train and test SSBs for prediction. In other words, we predict the types of proteins with either a hierarchical approach (Fig. 1A) or a multi-class approach that simultaneously predicts SSBs, DSBs, RBPs, and non-NABPs for any given protein (Fig. 1B). The other major difference is that we use ESM2, a protein language model pretrained on a large number of protein sequences with 15 billion parameters (Lin *et al.* 2023), to extract features for training and testing. Protein language models are trained with deep neural networks for representing each protein sequence and have shown much improved performance in different types of bioinformatics studies, such as protein structure prediction (Jing *et al.* 2024, Lin *et al.* 2023), protein localization prediction (Luo *et al.* 2024), structure-based prediction of protein-nucleic acid binding sites (Roche *et al.* 2024, Sagendorf *et al.* 2024), and others (Avraham *et al.* 2023, Yeung *et al.* 2023, Rao *et al.* 2024).

Applying different types of classic machine learning methods, including SVM, RF, k-nearest neighbors (KNN), multilayer perceptron (MLP), and linear regression (LR), we demonstrate that the prediction accuracy for each step of the hierarchical approach can achieve up to 95%, and the overall prediction accuracy is about 85% for the multi-class approach. Moreover, similar to our previous studies, each approach achieves a good prediction balance on different datasets. With the multi-class approach developed in this study and the hierarchical approach from this study combined with the SSB/DSB and SSB/RBP classifiers from our recent work (Wu *et al.* 2024), we can achieve much improved prediction of NABPs for any given protein.

## 2 Methods

### 2.1 Datasets

We used the non-redundant datasets from our recent study for models training, validation and testing, which consist of 347 SSBs, 4211 DSBs, 5627 RBPs, and 12 899 non-NABPs after removing redundant entries using Cd-hit with a cutoff of 0.4 (Wu and Guo 2024). For the hierarchical approach, in the first step of non-NABP/NABP classification, we combined all the SSBs, DSBs, and RBPs as the NABP set, and randomly selected 79% non-NABPs as the non-NABP set; in the second step of DBP/RBP classification, all the SSBs and DSBs are combined as the DBP group, and 81% RBPs are randomly selected as the RBP group (Fig. 1A). For the multi-class approach, we used all the SSBs as the SSB group, randomly selected 8.3% DSBs, 6.2% RBPs, and 2.7% non-NABPs as the DSB, RBP, and non-NABP sets, respectively (Fig. 1B). For each prediction, we randomly selected 70% as the training set, and the remaining 30% as the testing set. Within the training set, a five-fold cross-validation strategy was employed to select the best hyperparameters for each model. To make sure that the prediction performance is not biased from a specific training/testing split of the dataset, we carried out 100 independent prediction tests. In each independent prediction, the dataset was randomly split into training (70%) and testing (30%), then the training, validation, and testing were carried out and the performance values (see Section 2.4) were recorded. The means and standard deviations (SDs) were then calculated from these 100 independent tests.

### 2.2 Features extracted from ESM2

We downloaded the pretrained esm2_t33_650M_UR50D from ESM2 (Lin *et al.* 2023) for extracting the embedded features. For each protein, we first extracted its tokens using the pretrained alphabet function from esm2_t33_650M_UR50D, then derived the per-residue representations using the tokens of the protein using the trained model with repr_layers equal to 33. The per-sequence representations were then extracted by averaging the per-residue representations. For each protein, the per-sequence representations have the same length, a vector of 1280 values.

### 2.3 Machine learning models

Five machine learning models, SVM, MLP, KNN, LR, and RF were applied for training and testing. Classifiers SVC, MLPClassifier, KNeighborsClassifier, LogisticRegression, and RandomForestClassifier from sklearn (https://scikit-learn.org/stable/api/index.html) were used for SVM, MLP, KNN, LR, and RF, respectively. For each model, we used GridSearchCV with the default five-fold cross-validation to tune and select the best hyperparameters. The hyperparameters used in each machine learning model are summarized in https://github.com/unccguolab/Prediction-of-nucleic-acid-binding-proteins-using-protein-language-model.

### 2.4 Evaluation metrics

Five different evaluation metrics were applied to assess the performance of each model: accuracy (ACC), sensitivity or recall (SN/REC), specificity (SP), Matthews correlation coefficient (MCC), and F1-Score (F1) (1–6). ACC calculates the number of correctly predicted cases over the total cases. SN/REC represents number of correctly predicted positive samples over the total positive samples. SP is the ratio of the correctly predicted negative samples over the total negative samples. F1 considers both false positives and false negatives while MCC offers more balanced assessment of the overall performance.


(1) $$\mathrm{ACC}=\frac{\mathrm{TP}+\mathrm{TN}}{\mathrm{TP}+\mathrm{FN}+\mathrm{TN}+\mathrm{FP}}.$$

(2) $$\mathrm{SN}\left(\mathrm{REC}\right)=\frac{\mathrm{TP}}{\mathrm{TP}+\mathrm{FN}}.$$

(3) $$\mathrm{SP}=\frac{\mathrm{TN}}{\mathrm{TN}+\mathrm{FP}}.$$

(4) $$\mathrm{MCC}=\frac{\left(\mathrm{TN}\times \mathrm{TP}\right)-(\mathrm{FN}\times \mathrm{FP})}{\sqrt{\left(\mathrm{TP}+\mathrm{FN})(\mathrm{TP}+\mathrm{FP})(\mathrm{TN}+\mathrm{FN})(\mathrm{TN}+\mathrm{FP}\right)}}.$$

(5) $$\mathrm{PRE}=\frac{\mathrm{TP}}{\mathrm{TP}+\mathrm{FP}}.$$

(6) $$F1=\frac{2\times \mathrm{REC}\times \mathrm{PRE}}{\mathrm{REC}+\mathrm{PRE}}.$$

where TP, TN, FP, and FN represent true positive, true negative, false positive and false negative, respectively.

## 3 Results

### 3.1 Hierarchical approach

By applying the features extracted from ESM2 to different machine learning models, high prediction accuracy is observed in each step of the hierarchical approach. For prediction between non-NABPs and NABPs (Fig. 1A), all five models achieve greater than 90% overall prediction accuracy on the testing datasets with MLP, SVM, and LR having comparable and better performance than KNN and RF (Table 1). MLP (ACC = 94.3%, SN = 94.37%, SP = 94.22%, MCC = 0.886, F1 = 94.3%) performs slightly better than SVM and LR. The best performance from the SVM prediction uses a linear kernel as shown in the linked hyperparameters file (see Section 2), which is not surprising since a simple LR model can achieve similar high prediction accuracy. Notably, all three top performing machine learning models, SVM, MLP, and LR show a balanced sensitivity and specificity with very low SDs (Table 1).

**Table 1.** Performance evaluation on testing datasets for the hierarchical approach.^a^

Classifier	Methods	ACC (%)	SN (%)	SP (%)	MCC	F1 (%)
Non-NABP/NABP	SVM	93.79 ± 0.29	94.19 ± 0.44	93.38 ± 0.48	0.876 ± 0.006	93.81 ± 0.29
	MLP	**94.30 ± 0.38**	**94.37 ± 0.84**	**94.22 ± 0.78**	**0.886 ± 0.008**	**94.30 ± 0.39**
	KNN	91.31 ± 0.29	94.21 ± 0.48	88.41 ± 0.58	0.828 ± 0.006	91.56 ± 0.28
	LR	93.30 ± 0.28	93.98 ± 0.45	92.61 ± 0.48	0.866 ± 0.006	93.35 ± 0.28
	RF	90.35 ± 0.40	91.20 ± 0.64	89.50 ± 0.60	0.807 ± 0.008	90.43 ± 0.40
DBP/RBP	SVM	**95.54 ± 0.35**	94.72 ± 0.67	**96.36 ± 0.49**	**0.911 ± 0.007**	**95.50 ± 0.36**
	MLP	95.49 ± 0.36	**95.30 ± 0.66**	95.68 ± 0.72	0.910 ± 0.007	95.48 ± 0.36
	KNN	92.23 ± 0.44	92.95 ± 0.71	91.51 ± 0.70	0.845 ± 0.009	92.29 ± 0.44
	LR	94.82 ± 0.38	93.95 ± 0.74	95.69 ± 0.55	0.897 ± 0.008	94.78 ± 0.39
	RF	90.21 ± 0.44	86.71 ± 0.83	93.71 ± 0.73	0.806 ± 0.009	89.86 ± 0.48

a The data shown in the table are the means and SDs from 100 independent rounds. The bold numbers represent the best performance in each classifier and each evaluation approach.

**Figure 2.**
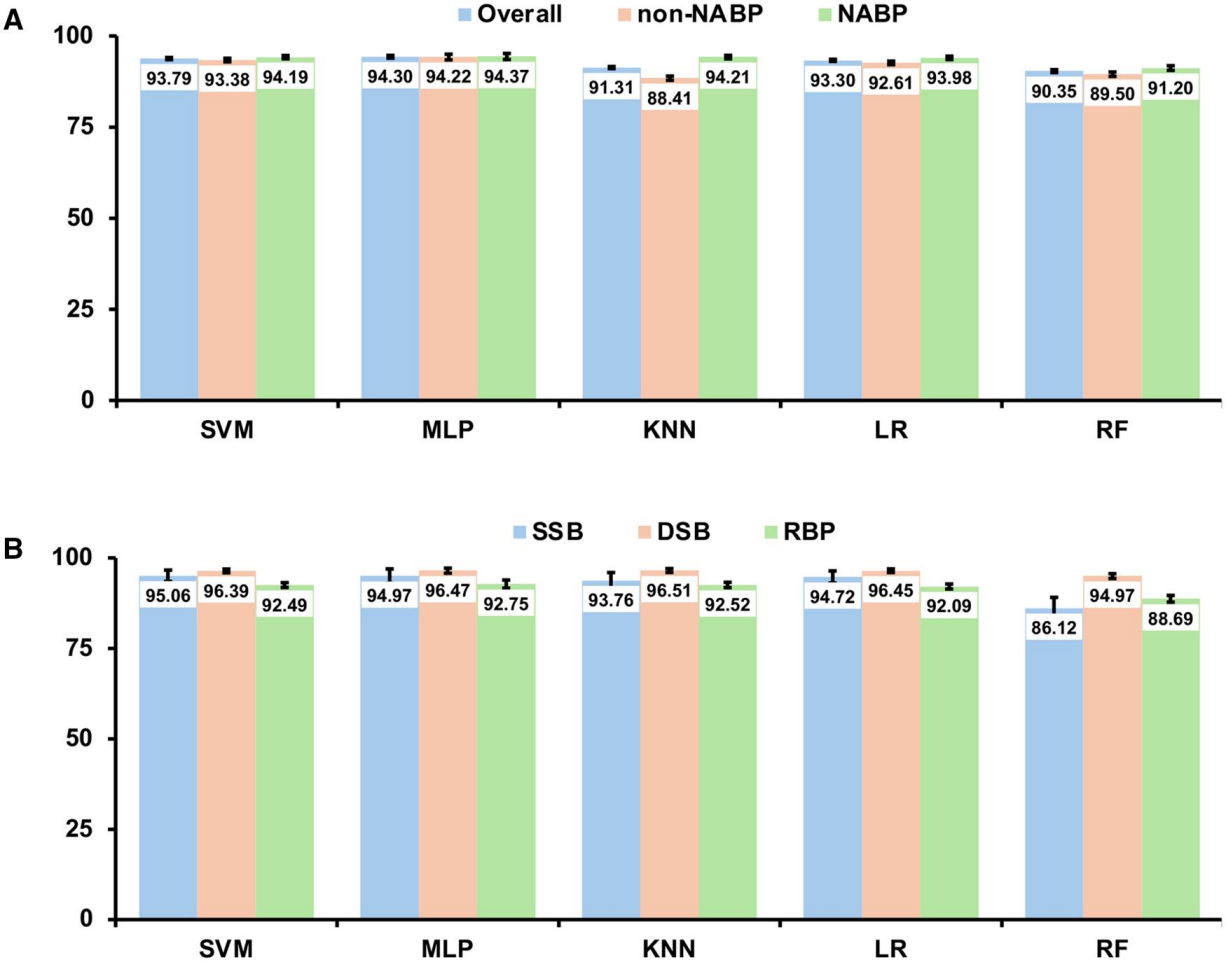
Prediction accuracy of non-NABP/NABP classification on the testing dataset. (A) Prediction accuracy on the overall, non-NABP and NABP datasets. (B) Prediction accuracy on the individual SSB, DSB, and RBP datasets.

The performance of each model on individual datasets, including non-NABPs and NABPs, has also been evaluated. As shown in Fig. 2A, except for KNN (88.41% for non-NABPs versus 94.21% for NABPs), all other models have similar prediction accuracy between non-NABPs and NABPs. The prediction accuracy for each of the three individual types of NABPs, SSBs, DSBs, and RBPs, was also assessed. Except for the RF model, the prediction accuracies among these three different types of NABPs are also comparable with a similar pattern: DSB has the best prediction accuracy followed by SSB and RBP (Fig. 2B).

For the DBP/RBP classification step (Fig. 1A), SVM, MLP and LR are the top performers with SVM having a slightly better performance (ACC = 95.54%, SN = 94.72%, SP = 96.36%, MCC = 0.911, F1 = 95.5%) (Table 1). Similar to the non-NABP/NABP prediction, each of the three top models has similar sensitivity and specificity with very small SDs for DBP/RBP classification (Table 1). RF has the largest difference between sensitivity (86.71%) and specificity (93.71%) (Table 1). While there are no non-NABP/NABP classifiers that we can compare directly, we compared our SVM-based DBP/RBP classifier with a number of DBP/RBP predictors that have been published and available for testing (Kumar *et al.* 2007, 2011) (Zhang and Liu 2017, Rahman *et al.* 2018, Zheng *et al.* 2018, Zhang *et al.* 2021, Pradhan *et al.* 2023a,b, Wu and Guo 2024). As we reported in our recent study, the negative datasets used in these predictions are either non-DBP (for DBP predictors) or non-RBP (for RBP predictors) (Wu and Guo 2024). In other words, the non-DBP datasets include both non-NABPs and RBPs and the non-RBP datasets consist of non-NABPs and DBPs. For the purpose of fair comparisons, we generated the negative datasets to mimic the published compositions using the entries from our newly created dataset and tested the performances of these programs (Wu and Guo 2024). Table 2 shows that our SVM predictor with ESM2 derived feature outperforms these tested DBP/RBP predictors.

**Table 2.** Performance comparison with other DBP/RBP predictors.

Predictors^*^	Prediction types	Methods used	REC	PRE	F1	Neg. Set ACC	Total ACC
DNAbinder^a^	DBP	SVM	0.72	0.5	0.59	0.27	0.49
DPP-PseAAC^b^	DBP	RF	0.47	0.52	0.49	0.57	0.52
PlDBPred^c^	DBP	ADB	0.65	0.75	0.7	0.78	0.71
RNAPred^d^	RBP	SVM	0.82	0.57	0.67	0.38	0.6
DeepRBPPred (balance)^e^	RBP	CNN	0.88	0.55	0.68	0.28	0.58
DeepRBPPred (unbalance)^e^	RBP	CNN	0.76	0.55	0.64	0.38	0.57
RBPLight^f^	RBP	LGB	0.72	0.69	0.7	0.67	0.7
DeepDRBP-2L^g^	DBP/RBP	CNN+LSTM	0.88	0.76	0.81	0.72	0.8
DBP/RBP (our previous study)^h^	DBP/RBP	CNN+LSTM	0.81	0.81	0.81	0.82	0.81
DBP/RBP (this study)	DBP/RBP	SVM	**0.95**	**0.96**	**0.96**	**0.95**	**0.96**

* a: Kumar *et al.* (2007); b: Rahman *et al.* (2018); c: Pradhan *et al.* (2023a, 2023b); d: Kumar *et al.* (2011); e: Zheng *et al.* (2018); f: Pradhan *et al.* (2023a,b); g: Zhang *et al.* (2021); h: Wu and Guo (2024).

Bold numbers represent the best performance in each category.

**Figure 3.**
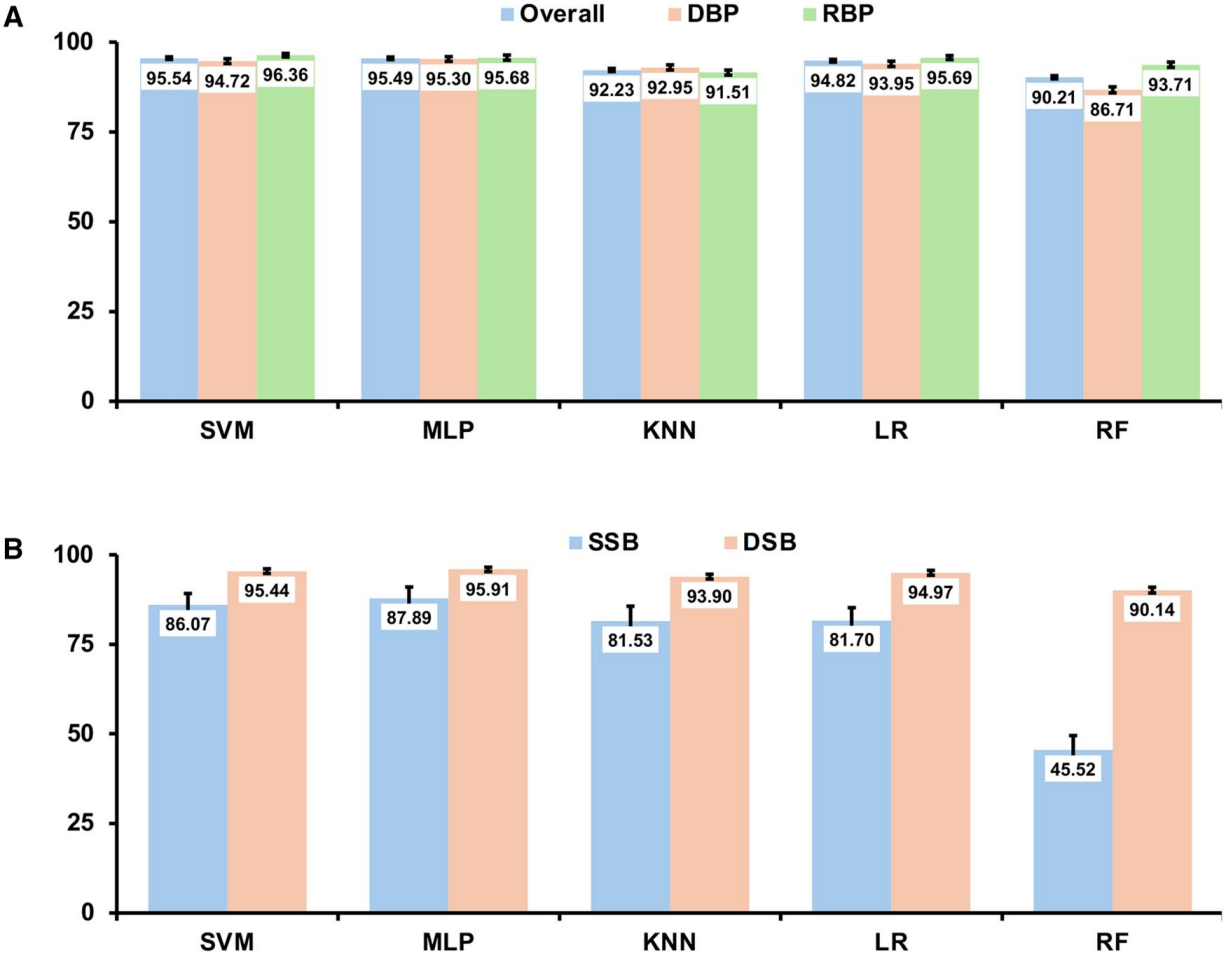
Prediction accuracy of DBP/RBP classification on testing datasets. (A) Prediction accuracy on the overall, DBP, and RBP datasets. (B) Prediction accuracy on the individual SSB and DSB datasets.

When checking the individual prediction accuracy for DBPs and RBPs, we found that DBPs and RBPs have similar prediction accuracy except for the RF model (Fig. 3A), suggesting that these models have good balance between the DBP and RBP datasets. Between the two different types of DBPs, the prediction accuracy of SSBs is relatively lower than that of DSBs from all the five models, especially for RF with only about 45% prediction accuracy on SSBs (Fig. 3B). But all four other machine learning models achieve over 81% prediction accuracy, much higher than that (∼40%) in our recently developed deep learning model (Wu and Guo 2024).

### 3.2 Prediction of non-NABPs, SSBs, DSBs, and RBPs with a multi-class approach

**Figure 4.**
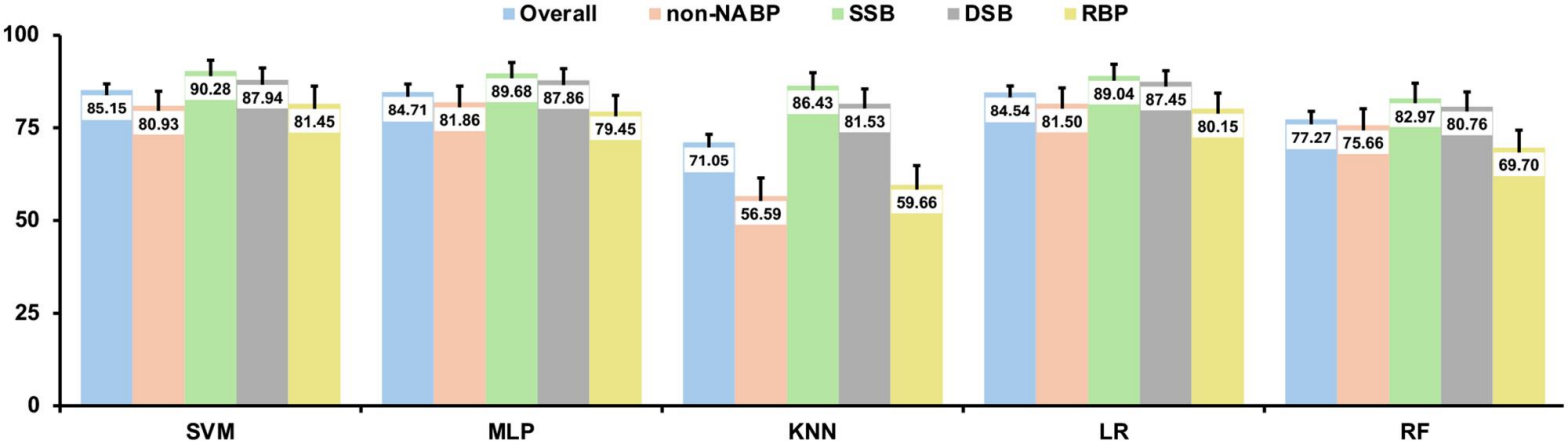
Prediction accuracy on the overall, non-NABP, SSB, DSB, and RBP testing datasets of the multi-class approach.

We also developed a multi-class approach for prediction of non-NABPs, SSBs, DSBs, and RBPs simultaneously. As shown in Fig. 4, similar to the hierarchical approach, the top three models are SVM, MLP, and LR, which have comparable overall prediction accuracy on the testing datasets with SVM model having the highest accuracy of 85.15%. KNN has the lowest overall prediction accuracy at 71.05%. In addition, compared to other four models, KNN has the largest variations among individual groups, ranging from the highest 86.43% (SSB) to the lowest 56.59% (non-NABP) (Fig. 4). One common pattern among all five models is that SSB has the best prediction accuracy.

For any given protein, the overall prediction accuracy between the hierarchical and multi-class approaches is quite comparable. Since the overall prediction accuracy for each individual type of proteins in the hierarchical approach is contingent on the previous steps, the overall prediction accuracy for each type of protein is a joint result from all three steps, non-NABP/NABP (93.79%), DBP/RBP (95.54%), and SSB/DSB (95.11% from our recently published report) (Wu *et al.* 2024). Therefore, the estimate of the overall prediction accuracy for each type of protein is about 93.79% × 95.54% × 95.11% = 85.23%, which is very similar to the overall prediction accuracy of the SVM multi-class approach (85.15%) (Fig. 4). However, performance on individual types of proteins may be different between the hierarchical and multi-class approaches. For example, the prediction of the SSBs has a higher prediction accuracy from the multi-class SVM approach (90.28%) than that from the hierarchical SVM approach (95.06% × 86.07% × 96.61% = 79.04%). As for DSBs, the prediction accuracy of the multi-class SVM approach (87.94%) is about 5% higher than the hierarchical SVM approach (92.49% × 95.44% × 93.6% = 82.62%). The third percentage values 96.61% and 93.6% from the above calculations are adopted from the SSB/DSB classification model from our recently published work (Wu *et al.* 2024). The prediction for non-NABPs and RBPs are the opposite of SSBs and DSBs in which the hierarchical approach has a better prediction accuracy than the multi-class model.

The prediction accuracy for non-NABPs is 93.38% based on the hierarchical SVM model (Fig. 2A) while the accuracy from the multi-class SVM model is only 80.93% (Fig. 4). For prediction of RBPs, the hierarchical model results in an overall accuracy of 89.12% (92.49% × 96.36%) (Figs. 2B and 3B) while the prediction accuracy is 81.45% from the SVM multi-class method (Figure 4). Therefore, depending on the purpose of applications, if the main goal is to predict novel SSBs or DSBs from proteins with unknown functions, the multi-class model is a better choice than the hierarchical approach.

## 4 Discussion

Although both DBP/RBP predictors and SSB/DSB predictors based on machine learning methods have been developed, the assumption that the target protein is either an NABP for DBP/RBP classification or a DBP for SSB/DSB classification makes these models less practically useful when a protein without any known functions is given. Therefore, a more practical approach is to develop programs that can accurately predict the different types of NABPs for any given protein. Our previously developed hierarchical and multi-class approaches for prediction of NABPs for any given protein without known function achieve an overall accuracy of about 72% (Wu and Guo 2024). Though they perform better compared to the published models, there is still room to improve.

In general, the performance of a machine learning model depends on three major factors, the datasets for training and testing, features used for training and testing, and machine learning models. The prediction accuracy seems to reach the limit when using the traditional sequence features such as PSSM and Hidden Markov Model (HMM) profiles. In our recent development of SSB/DSB and SSB/RBP classifiers, we also explored the addition of a structural feature, the predicted protein secondary structure types from DeepCNF (Wang *et al.* 2016). We demonstrated that even though the structural feature can help increase the prediction accuracy, the improvement is incremental at 2%–4% while the feature from protein language model ESM2 alone increases the performance dramatically (Wu *et al.* 2024). Protein language models learn from diverse sequences spanning the evolutionary tree and have proven to be powerful tools for sequence design, variant effect prediction, function and binding site prediction, and structure prediction (Avraham *et al.* 2023, Lin *et al.* 2023, Yeung *et al.* 2023, Jing *et al.* 2024, Luo *et al.* 2024, Rao *et al.* 2024, Roche *et al.* 2024, Sagendorf *et al.* 2024). Different protein language models have been developed in the past several years, including Bidirectional Encoder Representations from Transformers (BERT) and ProteinBert, a universal deep-learning model of protein sequence and function, pretrained on ∼106M proteins (Devlin *et al.* 2019). While ProteinBert performs well in representing features for any given protein (data not published), since it uses both sequences and GO annotations to pretrain models, it is not suitable for protein function prediction. ESM2, an evolutionary-scale predictor, on the other hand, only uses protein sequences for training (Lin *et al.* 2023). A recent study revealed the power of the protein language model ESM2 on protein-nucleic acid binding site prediction (Roche *et al.* 2024). The ablation study demonstrated that without ESM2, there is a big drop of prediction performance. However, discarding both of the evolutionary features, PSSM and MSA (for multiple sequence alignment), only results in a very small decrease of performance (Roche *et al.* 2024).

To improve the prediction accuracy, in this study we developed a hierarchical approach and a multi-class approach by exploring features from ESM2 and the newly developed datasets from our group. By applying the features from ESM2, our machine learning models, especially SVM, MLP, and LR achieve very high prediction accuracy, up to 95% for each step of the hierarchical approach and 85% for the multi-class approach, suggesting an overall prediction accuracy increase of 13% (85% versus 72%) over the non-ESM2 predictions for any given proteins without known functions. By comparing the overall prediction accuracy from the hierarchical and the multi-class approaches, we found that the multi-class SVM model is suitable for predicting novel SSBs and DSBs with higher accuracy while the hierarchical method can predict non-NABPs and RBPs better than the multi-class approach.

## Data Availability

The datasets and code can be found at https://figshare.com/projects/Prediction_of_nucleic_acid_binding_proteins_using_protein_language_model/211555.
